# A Lipase Gene of *Thermomyces lanuginosus*: Sequence Analysis and High-Efficiency Expression in *Pichia pastoris*

**DOI:** 10.3390/ijms252111591

**Published:** 2024-10-29

**Authors:** Le Yi, Lifeng Cheng, Qi Yang, Wei Luo, Shengwen Duan

**Affiliations:** 1 Institute of Bast Fiber Crops, Chinese Academy of Agricultural Science, No. 348 Xianjia Road, Changsha 410205, China; 2 Key Laboratory of Carbohyrate Chemistry and Biotechnology, Jiangnan University, Ministry of Education, No. 1800 Lihu Road, Wuxi 214122, China

**Keywords:** *Thermomyces lanuginosus*, *Pichia pastoris*, lipase, heterologous expression, bioinformatics analysis

## Abstract

Lipase, a type of enzyme that decomposes and synthesizes triglycerides, plays an important role in lipid processing. In this study, a heat-resisting lipase gene (*lip*4) from *Thermomyces lanuginosus* was subcloned into the pPICZαA vector and then transformed into *Pichia pastoris* X33. The recombinant yeast cell concentration reached the maximum (119.5 g/L) at 144 h, and the lipase (Lip4) activity reached the maximum (3900 U/mL) at 168 h in 10 L bioreactor. Through bioinformatics analysis, S168, as the key site of Lip4, participated in the formation of the catalytic triads S168-D223-H280 and G166-H167-S168-L169-G170. Furthermore, S168 and seven conserved amino acids of G104/288, S105, A195, P196, V225 and I287 constitute the active center of Lip4. Specifically, the structure modeling showed two α-helices of the lid domain, outside the active pocket domain, controlling the entry of the substrate on Lip4. The potential glycosylation of Asn-33 may be involved in exhibiting the high stable temperature for lipase activity. Therefore, the eukaryotic system was constructed to express Lip4 efficiently, and the amino acid sites related to the catalytic efficiency of Lip4 were clarified, providing a new way for its subsequent property research and industrial application.

## 1. Introduction

Lipase [E.C. 3.1.1.3], also called triacylglycerol acylhydrolase, is a hydrolytic enzyme which acts on carboxylate bond. It releases diglycerol, monoglycerol, glycerol, and fatty acids by catalyzing hydrolysis of ester bonds of triacylglycerol [1,2]. Compared with chemical catalysis, lipase catalysis is environmentally friendly and sustainable and can be widely used in textile, paper, food, bioenergy, chemical, and detergent industries [3]. Traditionally, lipase has been obtained from animal pancreas and used as a digestive supplement in its crude or purified form [4]. However, compared to lipases in animals and plants, lipases of microorganisms have wide substrate specificity, a wider range of operating temperature and pH, and have been used to improve dietary fat digestion in patients with congenital pancreatic triglyceride lipase deficiency [5]. Moreover, they are used to decompose highly diverse racemic mixtures to produce important enantiomer drugs such as (R)-glycinol [6], ibuprofen [7], and so on.

The first commercially available recombinant lipase obtained from the fungus *T. lanuginosus* has been expressed in *Aspergillus niger* [8], large-scale lipase production has come from genera including *Bacillus* [9,10], *Pseudomonas*, *Staphylococcus*, etc. [11,12]. However, the existing production of lipase cannot meet the continuously increasing growth of the bioindustry [13]. The production cost and enzymatic characteristics of lipase are the main constraints against the commercialization of the lipase catalysis technique [14]. So far, many strategies have been developed to optimize the production of lipase in order to meet commercial demands, including improving enzyme production in a new system [15], improving purification efficiency and effect by molecular modification [16], studying the structure-function relations (such as high temperature, pH, and activity and stability in the organic solvent environment) and catalytic mechanism by using the point mutagenesis [17,18,19,20].

According to previous reports, lipase from *T. lanuginosus* has good application characteristics [21]. We successfully achieved the efficient expression of *lip*4 from *T. lanuginosus* by constructing a eukaryotic expression system and analyzed its derived protein 3D structure, which provided a new strategy for further study on the properties and functions of lipase Lip4 and its molecular modification.

## 2. Results and Analysis

### 2.1. Sequence and Structure Analysis of Lipase Lip4

**Figure 1:**
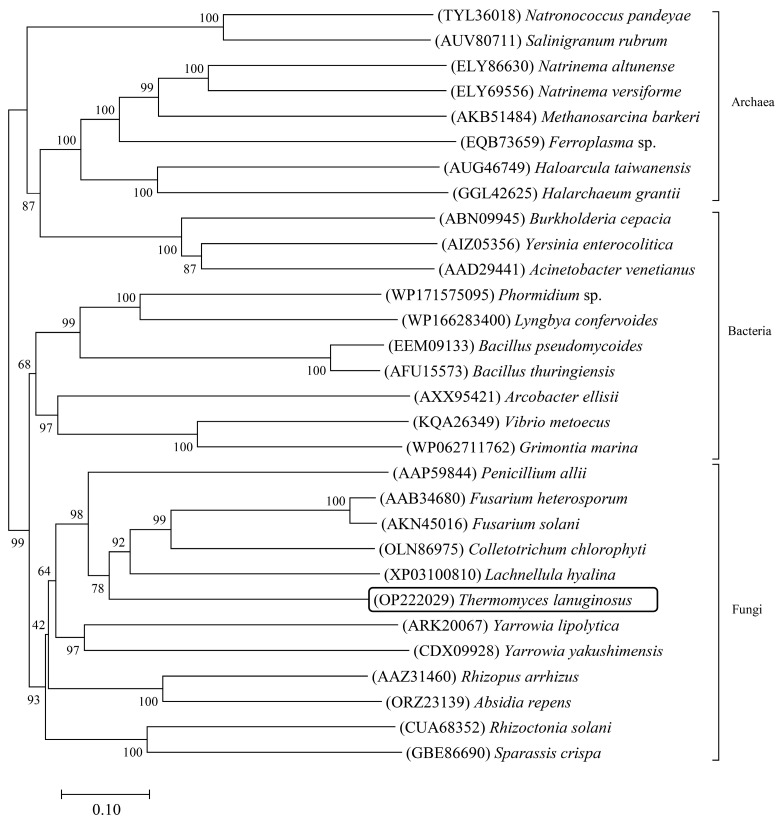
Phylogenetic tree constructed showing phylogenetic relationships among Lip4 and other lipases from microbiology. The tree analysis used the Neighbor-Joining (NJ) method by MEGA X, 1000 bootstrap replicates, and Kimura 2-parameter model for the substitution model. Scale bar at bottom indicates 0.10 nucleotide substitutions per site.

**Figure 2:**
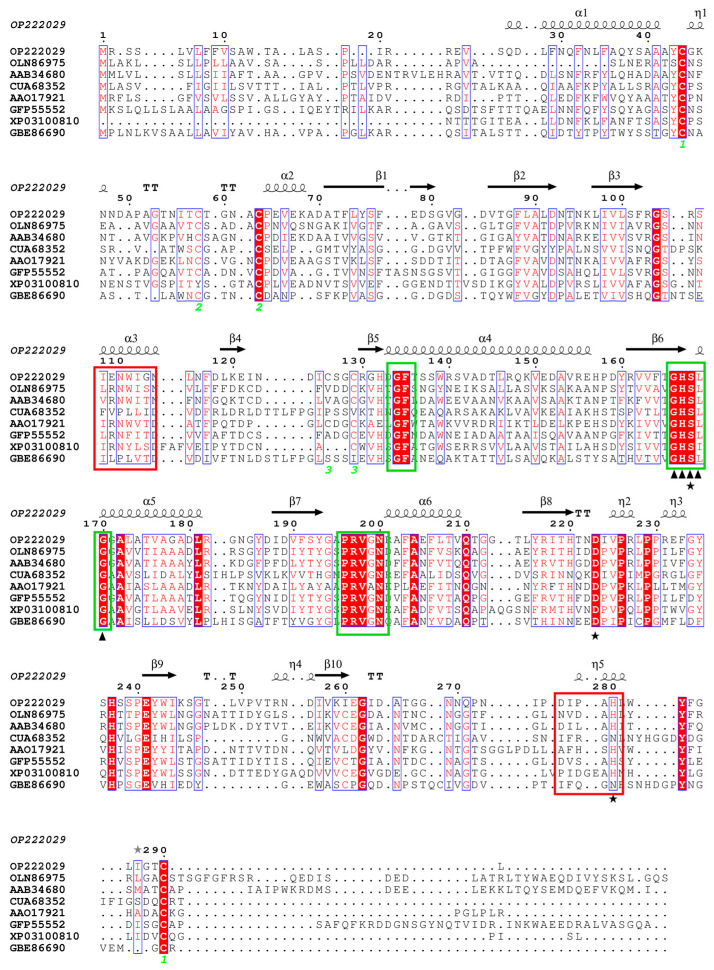
Homologous sequence alignment of Lip4 with lipases from other fungi sources. Note: Pentagram is marked as a triplet structure site. The triangle is the pentapeptide sequence at the active center. The red box is labeled with the sequence of lipase lid structures. The green boxes are marked as conservative sequences. The green numbers are labeled as cysteine pairs involved in forming disulfide bonds.

A phylogenetic tree was constructed by Lip4 and other lipase sequences from microorganisms in GenBank, and lipases showed typical species specificity (Figure 1). According to multi-sequence alignment, 6 cysteine of lipase Lip4 form three pairs of disulfide bonds, and conserved peptide acid segments are formed at G134-F135, G166-H167-S168-L169-G170, and P196-R197-V198-G199-N200 (Figure 2).

**Figure 3:**
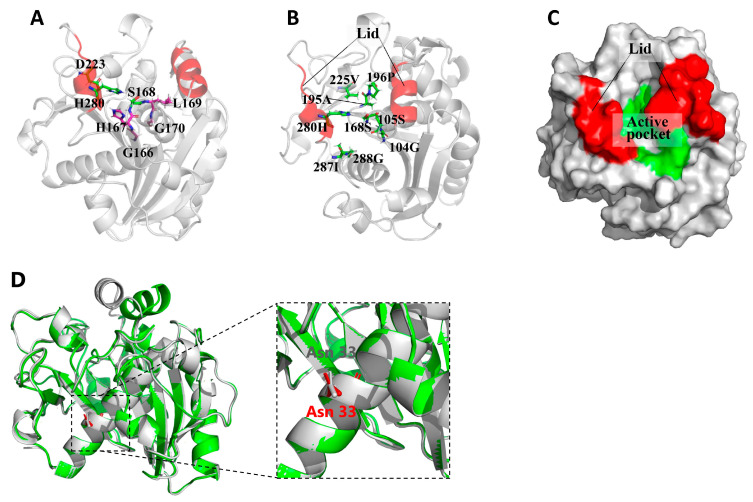
Structural modeling and active site illustration of lipase Lip4. (**A**) Catalytic triad; (**B**) Active center; (**C**) “Pocket” domain and “Lid” domain; (**D**) Potential glycosylation site of Asn-33.

The three-dimensional structure of lipase Lip4 was predicted to find the key amino acid sites or peptide segments. The results showed S168, D223, and H280 formed the triplet structure of lipase Lip4. The serine and other four peptide acid residues form the pentapeptide structure G166-H167-S168-L169-G170, which plays an important role in the catalysis function of lipase (Figure 3A). Serine and other seven conserved amino acids (G104/288, S105, A195, P196, V225 and I287) form the active center of lipase Lip4 (Figure 3B). The lid structure on the surface of the substrate binding pocket is composed of two α-helics, which can control the substrate entering the active pocket and binding to the catalytic site (Figure 3C). The potential glycosylation of Asn-33 may be involved in resistant heat for lipase Lip4 (Figure 3D).

### 2.2. Lipase Gene lip4 Expression in P. pastoris

**Figure 4:**
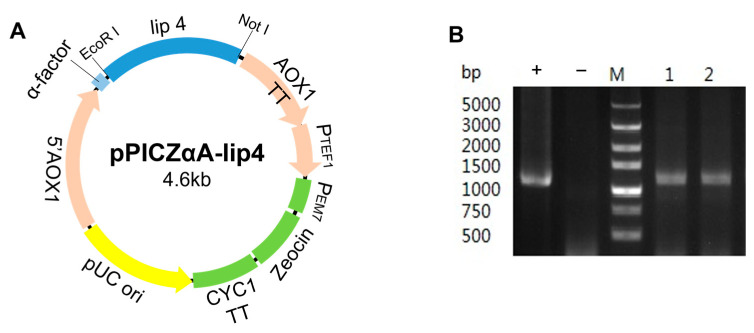
Construction of recombinant plasmid. (**A**) Recombinant plasmid lip4-pPICZαA; (**B**) PCR amplification of lipase gene lip4. M: Marker; 1–2: Lipase genes *lip*4 amplified using recombinants as templates; −: Negative control (ddH_2_O as template); +: Positive control (a known DNA fragment matched by the kit as template).

The cDNA of *T. lanuginosus* was used as the template and the designed primers were applied to PCR amplification of lipase gene *lip*4. This target fragment was purified and subcloned to the expression vector pPICZαA. The linearized recombinant plasmid *lip4*-pPICZαA was transformed into Electro-competent *P. pastoris* X33, and recombinant strains lip4-pPICZαA/GS115 were obtained (Figure 4A). Lipase gene *lip*4 was verified using the universal primer AOX1 with recombinant genomic DNA as a template (Figure 4B). The extracellular lipase activity of pPICZαA-*lip4*/X33 in shake flask fermentation can reach 423–500 U/mL.

**Figure 5:**
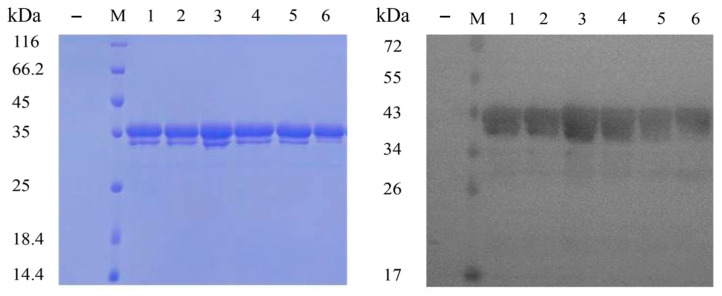
Analysis of lipase Lip4 secretory expression and purification by SDS-PAGE and Western blot. M: Protein molecular weight marker; 1–6: lipase Lip4 by ammonium sulfate precipitation and Ni-NTA affinity chromatography from the fermented liquid of engineering strain pPICZαA-*lip*4/X33 (induced); −: Negative control (not induced).

Fermentation broth was collected and centrifuged to obtain the extracellular crude lipase Lip4. The crude lipase Lip4 is purified through ammonium sulfate precipitation, followed by nickel column affinity chromatography, final detection is conducted using SDS-PAGE and western blotting (Figure 5). The results showed molecular weight of recombinant lipase Lip4 expressed after induction was about 35 kDa, slightly higher than the predicted molecular weight, which may be caused by adding 6×His tag into the recombinant lipase Lip4 or protein modification, such as glycosylation, phosphorylation, etc.

### 2.3. Lipase Production in Bioreactor

**Figure 6:**
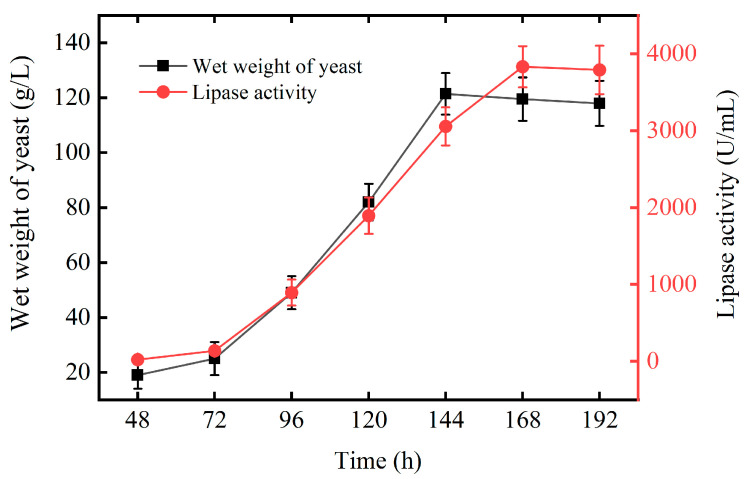
Growth and enzyme production patterns of recombinant yeast. The data were measured three times at described assay. Error bars in the figure indicate the standard error of three repetitions by software SPSS 19.0.

An initial fermentation volume of 4 L was conducted in a 10 L bioreactor, and the key parameters, such as dissolved oxygen and feeding, were strictly controlled. The wet weight and extracellular lipase activity of recombinant *P. pastoris* were detected in the fermentation of 192 h (Figure 6). The results showed that the wet weight and extracellular lipase activity of recombinant *P. pastoris* increased with the prolongation of fermentation time; the wet weight of pPICZαA-lip4/X33 reached the maximum, 119.5 g/L in the fermentation of 144 h and the extracellular lipase activity reached a peak of 3900 U/mL in the fermentation of 168 h.

### 2.4. Temperature Features of Lipase Lip4

**Figure 7:**
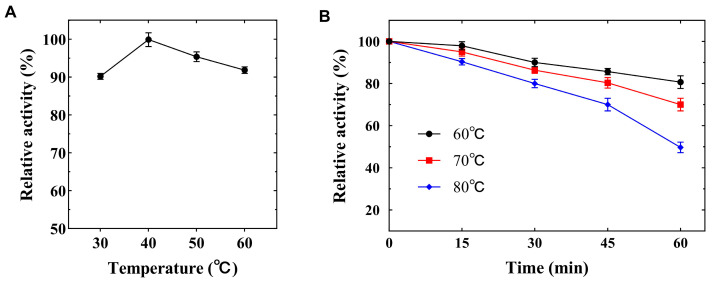
(**A**) Optimal temperature; (**B**) Thermo stability. The data were measured three times at given assay. Error bars in the figure indicate the standard error of three repetitions by software SPSS 19.0.

The optimal temperature of Lip4 was assessed across a range of temperatures (30 to 60 °C) using pH 8.8 100 mM Tris-Cl buffer at described assay conditions. The temperature optima were 40 °C for Lip4 (Figure 7A). The Lip4 was stable in a range of temperature from 60 °C to 70 °C for 30 min, with a residual activity of 80% at pH 8.8, while it exhibited a residual lipase activity of 80% in temperatures less than 60 °C for 60 min (Figure 7B).

## 3. Discussions

Enzymatic hydrolysis of lipase, with the advantage of selective esterification, hydrolysis, and transesterification, has become a preferred method for the industrial process of fatty acids due to the environmental protection, mild reaction conditions, and specificity [22]. Interestingly, the lipase from *T. lanuginosus* is a potential thermostability lipase, and it can facilitate enzymatic hydrolysis under high temperatures, decreasing the viscosity of substrate and increasing the solubility of substrate, diffusion coefficient, and reaction rate [21,23]. Thus, it can be inferred that lipase Lip4 is a multipurpose biological catalyst given a favorable vision in meeting the needs for several industries such as biodiesel, foods and drinks, leather, textiles, detergents, pharmaceuticals, and medicals [6].

Methylotrophic yeast *P. pastoris* has been the most common recombinant host system for the commercial production of heterologous proteins. Its expression is driven by one of the strongest known regulated promoters, the alcohol oxidase I (AOX1) promoter, induced by methanol. Furthermore, this system is able to achieve extremely high cell densities, enabling efficient protein production and secretion performing eukaryotic posttranslational modifications [24]. The finding of this study emphasized that Lipase Lip4 activity expressed by *P. pastoris* X33 had reached 3900 U/mL, which is higher than that from natural strain *T. lanuginosus* VAPS25 (18.85 U/mL) [17].

The potential glycosylation of Asn-33 may be involved in exhibiting the high stable temperature for lipase activity (Figure 3D). It was reported lipase gene from *T. lanuginosus* expressed in *P. pastoris* is the same as that expressed in *A. oryzae*, and confirmed that the Asn-33 site is conserved for glycosylation from *A. oryzae* to *P. pastoris* [25].

Lipase Lip4 from *T. lanuginosus* exhibited peak activity at 40 °C. The optimal temperature of Lip4 is quite lower than other microbiological lipases (Table 1). The lipase activity of Lip4 increased with an increase in temperature from 30 °C to 40 °C, but a further increase in temperature adversely affected the lipase activity, which might be due to the denaturation of the enzyme at higher temperatures [26]. Lipase Lip4 exhibits thermal stability at temperatures ranging from 60 °C to 80 °C for 60 min (Table 1), which is higher than that of *B. aerius* and *S. aurata* [26,27]. As compared to the described lipolytic enzymes, lipase Lip4 demonstrated noteworthy heat resistance. These findings underscore Lip4 as a robust thermo lipase, adept at functioning effectively at elevated temperatures encountered in industries. With valuable temperature features, Lip4 can enhance the substrate solubility reaction rates; reduce viscosity and contamination rates in various bioprocesses.

Interestingly, the purified SeLipC shows the highest activity at 60 °C, whereas its thermal stability is significantly improved when protein concentration is increased, as confirmed by thermal deactivation kinetics, circular dichroism, and differential scanning calorimetry [28]. It indicates that heat resistance is a relative parameter in the given circumstance. Therefore, we hypothesized that increasing the concentration and purity of the lipase Lip 4 may help extend the lipase’s active time and even its shelf life. This hypothesis will be confirmed in subsequent studies.

**Figure 8:**
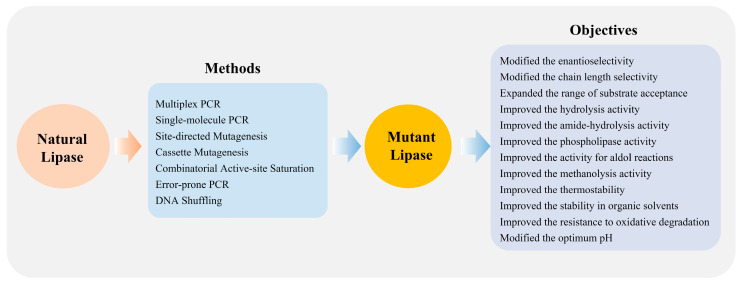
Modification of lipases to development of novel lipase by protein engineering.

Enzymatic characteristics of lipase, such as enzyme activity, optimum temperature, thermostability, pH stability and, substrate selectivity, etc., were improved by protein engineering measures [31,32]. Protein engineering measures introducing amino acid site mutations or peptide insertion/deletion involved in directed evolution, which is to simulate the Darwin evolutionary process under laboratory conditions, artificially introduce abundant mutations through random mutation and recombination, and construct the gene mutation library of target proteins (Figure 8). Lipase Lip4 is going to be modified according to its structural characteristics. The hydrophobicity and electrostatic area, where the substrate combines with the active pocket, will be changed to improve catalytic activity. The binding affinity of lipase Lip4 and substrate will be strengthened by replacing amino acid residues with stronger hydrophobicity and different charges in the following research. According to its structure and molecular docking results with different fatty acids, the relationship between charge changes and substrate bonding is disclosed. The improvement of thermostability observes the principle of increasing rigidity of local peptide fragments, and the thermostability of the whole lipase Lip4 is improved by introducing in the disulfide bond and tempering secondary structure. Furthermore, existing technical means are going to be improved by combining them with the catalytic characteristics of lipase Lip4.

## 4. Materials and Methods

### 4.1. Plasmid and Reagent

The expression vector pPICZαA, *P. pastoris* X33, methanol, SpeedyCut Sacl, Ni-NTA, and IgG were purchased from Sangon (Shanghai, China). *E. coli* DH5α, Ultra HiFidelity PCR Kit, RNAsimple total RNA extraction kit, TIANScriptⅡ RT Kit, agarose gel DNA recycle kit, and plasmid extraction kit were purchased from Tiangen (Beijing, China). The primer synthesis and DNA sequencing were completed by Tsingke Biotechnology Co., Ltd. (Changsha, China). Other pure biochemical reagents for analysis were purchased from Sinopharm Group (Shanghai, China).

### 4.2. Strain and Medium

*T. lanuginosus* FFM-01 was screened and preserved by the plant fiber functional material innovation team of the Institute of Bast Fiber Crops, Chinese Academy of Agricultural Sciences.

Luria-Bertani (LB) medium (5 g/L yeast extract, 10 g/L peptone, and 10 g/L sodium chloride) was used for culturing *E. coli* DH5α, and 100 μg/mL ampicillin was added for recombinant plasmid selection. PDA liquid medium (potato 20%, glucose 2%) was used to culture *T. lanuginosus.* YPD liquid medium (10 g/L yeast extract, 20 g/L peptone, and 20 g/L glucose) was used for culturing yeast. The yeast extract peptone dextrose medium (YPDS medium) consisted of 10 g/L yeast extract, 20 g/L peptone, 20 g/L glucose, 1 mol/L sorbitol, 20 g/L agar, and 100 μg/mL Zeocin. The Shake-flask fermentation medium (BMGY liquid medium) was composed of 10.0 g/L yeast extract, 20.0 g/L peptone, 10.0 g/L glycerol, 13.4 g/L YNB, 100 mmol PBS broth (pH 6.0), 0.016 μmol/L biotin. The inducing fermentation medium (BMMY liquid medium) was composed of 10.0 g/L yeast extract, 20.0 g/L peptone, 10.0 g/L methanol, 13.4 g/L YNB, 100 mmol PBS broth (pH 6.0), 0.016 μmol/L biotin.

### 4.3. Extraction of Total RNA from T. lanuginosus

*T*. *lanuginosus* FFM-01 was cultured in 50 mL of PDA liquid media at 50 °C, 150 r/min for 72 h, and then centrifuged to harvest microbiological cells. The total RNA was extracted according to the instruction of the RNAsimple total RNA extraction kit and then transformed into cDNA through RT-PCR according to the instruction of TIANScriptⅡ RT Kit (TianGen, Beijing, China). The cDNA was kept under −20 °C for use.

### 4.4. Lipase Gene Cloning and Expression

Lipase gene *lip*4 of *T. lanuginosus* FFM-01 (GenBank: OP222029) was amplified and cloned into the pPICZαA plasmid. The purified pPICZαA plasmid and the lipase gene *lip*4, digested by *Eco*R I and *Not* I, were connected by T_4_ DNA ligase overnight under 16 °C, then recombinant plasmids lip4-pPICZαA were transformed to *E. coli* DH5α through heat-shock method and coated onto the LB nutrition agar containing 100 μg/mL ampicillin, cultured in 37 °C for 24 h. Positive clones were screened and cultured in LB broth overnight to extract recombinant plasmids for PCR identification. The positive plasmids were submitted to Tsingke Biotechnology Co., Ltd (Changsha, China). for sequencing. Accurate recombinant plasmids were linearized with *Sac*Ⅰ and mixed with the competent cells of *P. pastoris* X33 at the volume ratio of 1:8. The mixture was transformed into a pre-cooled electric cup for 5 min of ice bath, followed by 5 ms of electric shock. Next, the pre-cooled sorbitol was added immediately. Additionally, the vector without lipase gene *lip*4 was transformed to *P. pastoris* X33 as a negative control group. The solution after electroporation was incubated at a constant temperature (30 °C) for 2 h and then centrifuged. The above solution was spread onto the YPDS nutrient agar medium (containing 100 μg/mL Zeocin) and cultured at 30 °C.

The recombinant yeast was inoculated into 5 mL YPD liquid medium, cultured at 37 °C, 200 r/min for 24 h, and then inoculated into 50 mL BMGY medium, cultured at 28.5 °C, 200 r/min for 24 h. The fermentation broth was centrifuged at 4 °C, 5000× *g* for 5 min, the yeast cells were collected and washed with sterile normal saline (0.9% NaCl) twice, and then re-suspended the yeast cells with BMMY medium so that the optical density of the microbial solution *OD*_600_ was about 1.0. And then induced in BMMY liquid medium at 28.5 °C, 200 r/min for 168 h, during which sterile methanol was added every 24 h to maintain the methanol concentration at 1.0%. Extracellular lipase activity of recombinant yeast lip4-pPICZαA/X33 in the shaker was determined.

### 4.5. Fermentation

The initial volume of the nutrient broth was 4 L in a 10 L bioreactor, with an initial temperature of 28 °C, and the pH was adjusted to 6.0 with ammonia water. The unit of enzyme activity (U) w. The recombinant yeast lip4-pPICZαA/X33 was inoculated with 10% (400 mL) of YPD and cultured at 28 °C for 24 h. After fermentation for 24 h, the glycerol in the basal medium was basically exhausted, and the glycerol-fed-batch phase began. After 8-12 h incubation, the *OD*_600_ was about 150–200, and the glycerol in the fermentation broth was almost exhausted when glycerol was stopped. The amount of dissolved oxygen increased to more than 50%. Methanol was fed to 12 mL micronutrient, and the methanol induction phase was initiated. During the fermentation process, the dissolved oxygen was ensured to exceed 20%. Under this condition, the fermentation time was 192 h, and the wet weight and lipase activity were measured by samples taken every 24 h. The total fermentation time was 192 h.

### 4.6. Lipase Activity Detection

The fermentation liquor of engineering strain pPICZαA-lip4/X33 induced by methyl alcohol was collected and centrifuged at the rate of 12,000 r/min for 5 min. Supernate (500 mL) was collected and purified by ammonium sulfate precipitation and Ni-NTA affinity chromatography sequentially. 20 μL 5× Loading Buffer was added to 80 μL purified Lip4, boiling in a water bath for 10 min. The protein secretory expression and purification were tested by SDS-PAGE and western blot [33]. The fermented liquid of engineering strain pPICZαA-lip4/X33 without introduction was treated in the same way and used as the negative control group.

Lipase activity was detected according to the *p*-nitrophenol (*p*-NP) method [34]. The *p*-nitrophenol phosphate was dissolved in isopropanol to prepare 1 mM of the substrate solution A. The enzyme solution was diluted to the solution B with appropriate concentration by using pH 8.8 100 mM Tris-Cl buffer. The solution A and solution B were mixed uniformly after pre-heating under 37 °C and then reacted for 5 min under 37 °C. After finishing the reaction, 100 μL 10% SDS was added into samples immediately to terminate the enzymatic reaction. The lipase activity unit was calculated according to the *p*-NP standard curve by testing the absorbance *OD*_380_ [35]. The unit enzyme activity (U) was defined as the enzyme volume required to release 1 μmol *p*-NP per minute.

The optimal temperature for Lip4 activity was determined at various temperatures (30 °C to 60 °C) at pH 8.8, and the highest lipase activity was taken as 100%.

Thermostability was determined by measuring the residual lipase activity of Lip4 after incubation at 60–80 °C (10 °C internal) for 60 min under pH 8.8 by the standard method. The pre-incubated Lip4 activity at 4 °C was taken as 100% [36].

### 4.7. Bioinformatics Analysis

The basic physical and chemical properties of Lip4 were analyzed by the bioinformatics website Expasy (https://web.expasy.org/protparam/, accessed on 11 September 2024), the signal peptides were predicted by SignalP-5.0 (http://www.cbs.dtu.dk/services/SignalP/, accessed on 16 August 2024), and the domains of Lip4 were analyzed by Pfam (http://Pfam.xfam.org/search/sequence, accessed on 16 August 2024).

Cluster analysis of lipase amino acid sequences was performed using the bioinformatics software Mega X (v10.1.8). The secondary structure of Lip4 was predicted by computer online software PSIPRED (http://bioinf.cs.ucl.ac.uk/psipred/, accessed on 16 August 2024); Homologous modeling was conducted by the Swiss Bioinformatics Center online tool SWISS-MODEL (https://swissmodel.expasy.org/, accessed on 16 August 2024) for Lip4, VMD was used for mapping analysis, and Verify-3D was used to score the model, the results indicate that the obtained model is reliable, with 90.33% of the residual average 3D-1D scores ≥ 0.1.

## 5. Conclusions

The heat-resisting lipase gene (*lip*4) from *T. lanuginosus* is cloned to the expression vector pPICZαA and then transformed into *P. pastoris* X33 for high-efficiency expression. The yeast cell concentration reaches the peak at 144 h of induction culture, and the lipase activity reaches the peak (3900 U/mL) at 168 h. Moreover, amino acid sequences and characterization structures revealed that S168, D223, and H280 formed the triplet structure of the Lip4. Specifically, serine and the other four residues formed the pentapeptide structure G166-H167-S168-L169-G170, playing an important role in the catalysis function of Lip4. Moreover, serine forms the active pocket with seven conserved amino acids, including G104/288, S105, A195, P196, V225, and I287. The lid domain on the surface of the active pocket can prevent the binding of substrate with catalytic sites by keeping it out of the active pocket.

## Figures and Tables

**Table 1:** Comparison of temperature features of lipase Lip4 with other reported microbial lipases.

Source	Optimal Temperature	Stable Temperature	Reference
*Thermomyces lanuginosus*	40 °C	70 °C (30 min) 60 °C (60 min)	This study
*Streptomyces exfoliatus*	60 °C	60 °C (low concentration); >70 °C (high concentration)	[28]
*Pseudomonas helmanticensis*	50 °C	80 °C	[29]
*Bacillus aerius*	55 °C	60 °C	[26]
*Sparus aurata*	50 °C	40 °C	[27]
*Geobacillus kaustophilus*	65 °C	>75 °C	[30]

## Data Availability

Data are contained within the article.
